# RUNX3 and T-Bet in Immunopathogenesis of Ankylosing Spondylitis—Novel Targets for Therapy?

**DOI:** 10.3389/fimmu.2018.03132

**Published:** 2019-01-10

**Authors:** Matteo Vecellio, Carla J. Cohen, Amity R. Roberts, Paul B. Wordsworth, Tony J. Kenna

**Affiliations:** ^1^Nuffield Department of Orthopaedics, Rheumatology and Musculoskeletal Sciences, University of Oxford, Oxford, United Kingdom; ^2^Oxford Musculoskeletal Biomedical Research Unit, National Institute for Health Research, Oxford, United Kingdom; ^3^Oxford Comprehensive Biomedical Research Centre, Botnar Research Centre, National Institute for Health Research, Nuffield Orthopaedic Centre, Oxford, United Kingdom; ^4^Dunn School of Pathology, University of Oxford, Oxford, United Kingdom; ^5^Translational Research Institute, Princess Alexandra Hospital, Brisbane, QLD, Australia; ^6^Faculty of Health, Institute of Health and Biomedical Innovation, Queensland University of Technology, Brisbane, QLD, Australia

**Keywords:** ankylosing spondylitis, inflammation, functional genomics, autoimmunity, therapy

## Abstract

Susceptibility to ankylosing spondylitis (AS) is polygenic with more than 100 genes identified to date. These include *HLA-B27* and the aminopeptidases (*ERAP1, ERAP2*, and *LNPEPS*), which are involved in antigen processing and presentation to T-cells, and several genes (*IL23R, IL6R, STAT3, JAK2, IL1R1/2, IL12B*, and *IL7R*) involved in IL23 driven pathways of inflammation. AS is also strongly associated with polymorphisms in two transcription factors, RUNX3 and T-bet (encoded by *TBX21*), which are important in T-cell development and function. The influence of these genes on the pathogenesis of AS and their potential for identifying drug targets is discussed here.

## Introduction: AS Genetics

Ankylosing Spondylitis (AS) is a chronic inflammatory enthesitis predominantly affecting the axial skeleton (1). It is often associated with other conditions, such as uveitis, psoriasis, and inflammatory bowel disease (IBD), with which it has significant genetic overlap (2–5). The strongest genetic association of AS is with the HLA-B27 immune response gene (6). About 85% of British AS cases carry *HLA-B27* (odds ratio ~60) but probably no more than ~5% of those who are *HLA-B27* positive actually develop the condition, suggesting that other genes are also involved. Twin studies also support polygenic susceptibility (7) and this has been amply confirmed by genome-wide association studies (GWAS) (8–10). More than 100 loci have already been implicated in AS but for most the mechanisms underlying the associations are unknown. One exception appears to be the multiple “hits” in genes involved in interleukin (IL)-23 driven pro-inflammatory pathways (e.g., *IL23R, IL12B, IL6R, TYK2, IL27R, IL1R2, IL1R1*, and *STAT3*) (9–11). In murine models of the disease, resident IL23R-expressing cells can be demonstrated at the entheses (stress-bearing fibrous or fibro cartilaginous attachment of connective tissues, such as tendons or ligaments to bone). Further, the main features of the disease can be recapitulated simply by inducing hepatic overexpression of IL-23 without any evidence of recruitment of additional cell types to the entheses (12).

## HLA-B27 and Aminopeptidases

Because HLA-B27 plays a key role in antigen presentation to CD8+ cytotoxic T-cells (13), it is plausible that the inflammation in AS might be mediated by HLA-B27-restricted CD8+ cytotoxic T-cells, perhaps generated in response to microbial infection (14). However, it is also possible that depressed CD8+ T-cell function might be responsible, which could account for certain animal models of spondyloarthropathy (SpA) that develop in the absence of CD8+ T-cells (15). Further, in some models of autoimmune demyelinating disease, such as multiple sclerosis, CD8+ T-cells can act as either effector or suppressor cells (16). Specifically, the absence of CD8+ T-cells can be associated with more severe chronic disease or with increased susceptibility to relapses (17).

One of the earliest and most exciting GWAS findings indicated strong association between AS and certain aminopeptidases, initially with *ERAP1* (endoplasmic reticulum aminopeptidase 1) but subsequently also with *ERAP2* and the related *LNPEP* (leucyl/cystinyl aminopeptidase) (9). Further, some of these associations were replicated in related conditions, including psoriasis and IBD (18, 19). *ERAP1* has the second strongest association with AS and displays a synergistic interaction with *HLA-B27* (18–21); the *ERAP1* association is lost in *HLA-B27* negative cases but a weaker association with *ERAP2* can be seen both in *HLA-B27* negative and positive cases (22). ERAP1 and other aminopeptidases appear to play a significant role in trimming peptides transported from the cytosol to the endoplasmic reticulum to optimal length (8 or 9 amino acids) for loading on HLA class I molecules (23, 24). We and others have shown that AS-associated ERAP1 variants are involved in dysregulated peptide trimming that profoundly affects the range of peptide antigens presented to the immune system (25–27).

The protective allele of the *ERAP1 rs30187* polymorphism is associated with reduced peptidase activity resulting in alterations in the HLA-interacting *peptidome* (24, 28) of both pathogen-derived and host-derived antigens compared to the high-risk allele. Theoretically small molecule inhibitors of ERAP1 might therefore be of interest in the treatment of AS.

## The Contribution of IL23R

*IL23R* (encoding the specific portion of the heterodimeric interleukin 23 receptor) was the first non-MHC gene to be associated with AS (29, 30). The primary *IL23R* association with AS (also psoriasis and IBD) is with *rs11209026*, a coding SNP in the cytoplasmic tail, which alters IL-23R signaling (31, 32). In addition, a second independent association signal has been identified in the intergenic region downstream of *IL23R* and upstream of *IL12RB2* (encoding the 130kd β2 chain of the IL-12 receptor) (10). Our group recently identified a putative enhancer in this region of independent association, where the *rs11209032* SNP it is likely to be the candidate causal SNP in this region. Allelic variation of *rs11209032* may influence Th1-cell numbers (33). Further work is necessary to explain the mechanisms for these important observations.

IL-23 has critical roles in the pathogenesis of autoimmunity: it induces the Th17 cell population with a unique inflammatory gene signature (*IL17A, IL17F, IL6, CSF2, TNF, CCL20, CCL22, IL1R1*, and *IL23R*) (34).

The multiple associations (*IL23R, IL6R, STAT3, JAK2, IL1R1/2, IL12B*, and *IL7R*) with AS in the IL-23-driven pathways played a prominent role in promoting the successful targeting of the pro-inflammatory cytokine IL17A with the therapeutic monoclonal antibody secukinumab (35). Recently it has also been demonstrated that it is possible to use epigenetic approaches to inhibit Th17 cell maturation (integral to the pathogenesis of SpA) by targeting the bromodomains of transcriptional co-activators (such as CBP and p300) that recognize acetylated lysine residues in the regulatory elements associated with pro-inflammatory genes (36). However, the precise mechanisms involved in these therapeutic approaches are not entirely straightforward. Thus, although secukinumab is highly effective in AS (37), recent clinical trials of risankizumab, which targets the p19 subunit of IL-23 have proved ineffective (38). It is possible that there is a pharmacokinetic reason for this failure but this seems relatively unlikely given that the drug seems to be effective in similar doses in psoriasis and IBD (39, 40). It would seem more likely that there are alternative pathways through which IL17 production is sustained other than purely through IL23-driven mechanisms. Analogous therapeutic differences have been noted previously in the failure of secukinumab to benefit those with IBD despite its obvious immunogenetic similarities to AS (41). Further studies, both basic and clinical, are needed to unpick the mechanistic differences between the outcomes of the secukinumab and risankizumab trials.

## GM-CSF: a Novel Target in AS

GM-CSF, a cytokine involved in hematopoiesis and in immune responses, has been recently identified as a promising target in AS therapy. GM-CSF and its receptor are overexpressed in the synovial joints of patients with immune-mediated inflammatory SpA (42–44). A unique subset of GM-CSF+ CD4 T-cells has been identified by transcriptomics that represents an effector population distinct from Th1 and Th17 cells (45). GM-CSF promotes joint damage by recruiting granulocyte and macrophage precursors from activated bone marrow adjacent to synovial joints and causes the release of pro-inflammatory chemokines such as CCL17 (46). These processes, which are expanded in the synovial fluid of patients with axial SpA, led by GM-CSF, also induce the destruction of the cartilage and the resorption of the bone, inducing several matrix metalloproteinases and the osteoclast activating factor—RANKL (Receptor Activator of Nuclear Factor Kappa-B Ligand) (47, 48).

The use of monoclonal antibodies to neutralize GM-CSF have been very effective in preventing the progression of joint damage and inflammation in arthritic mice (49) and long-term phase II studies in RA are very encouraging (50). Furthermore, based on the evidences discussed above, a clinical trial with antibody targeting GM-CSF has recently started in AS patients.

## Transcription Factors Associated With AS

Most DNA single nucleotide polymorphisms (SNP) associated with complex diseases like AS do not cause protein coding changes; the R381N polymorphism in the cytoplasmic tail of the IL23 receptor associated with susceptibility to AS and IBD is the exception rather than the rule in this regard (32). On the contrary, most genetic associations in such multifactorial conditions are in non-coding regions of the genome where they exert epigenetic regulatory effects (51). Such associations in intergenic regions are well described in AS, and in at least some cases, probably alter susceptibility to the disease by influencing gene expression levels. Disease associated polymorphisms might influence regulatory elements (enhancers, silencers, promoters) through changes in chromatin remodeling or transcription factor binding. SNPs in regulatory regions could affect gene expression through changes in transcription factors (TFs) recruitment, DNA methylation, histone modification or miRNA expression or binding.

### RUNX3

The AS association at the *RUNX3* locus (Runt-related transcription factor 3) provides a good example of this type of genetic regulatory influence (10). There is convincing evidence that *RUNX3* is associated with AS and other forms of SpA, including psoriatic arthritis (52). RUNX3 plays a prominent role in the development and differentiation of CD8+ T-cells, which have been implicated in the pathogenesis of AS (53, 54). Three other AS–associated genes (*EOMES, IL7R*, and *ZMIZ1*) impacting on variation in CD8+ lymphocyte counts were also identified by the International Genetics of Ankylosing Spondylitis (IGAS) consortium (10). The robust genetic associations with *RUNX3, IL7R*, and *EOMES* (eomesodermin), all of which impact on CD8+ T-cells differentiation, support the involvement of CD8 T-cell pathology in AS. However, the precise mechanisms involved are likely to be more complex than simple effects on T-cell numbers. Thus, although the risk haplotype at RUNX3 (*rs6600247*) is associated with lower CD8+ T cell counts the opposite is seen with the risk haplotype at *IL7R* (*rs991570*), suggesting involvement of an unknown mechanism related to the IL7R/RUNX3 pathway.

*RUNX3* also has other fundamental roles in many other cell types. Its deletion leads to the dysregulation of cells including neurons, chondrocytes, Th1 helper cells, dendritic cells and NK cells (55).

In particular, RUNX3 is downstream of the TGF-β signaling pathway and may play a key role in CD4+ T-cell differentiation, potentially driving the imbalance of Th17/Treg cell in AS (56). RUNX3 is also a pivotal TF for the function of Innate Lymphoid Cells 3 (ILC3) via driving the expression of RORγt and its downstream target aryl hydrocarbon receptor (AHR), in ILC3 cells. RUNX3 deletion increases the susceptibility of ILCs to infection with *Citrobacter rodentium* (57).

**Figure 1:**
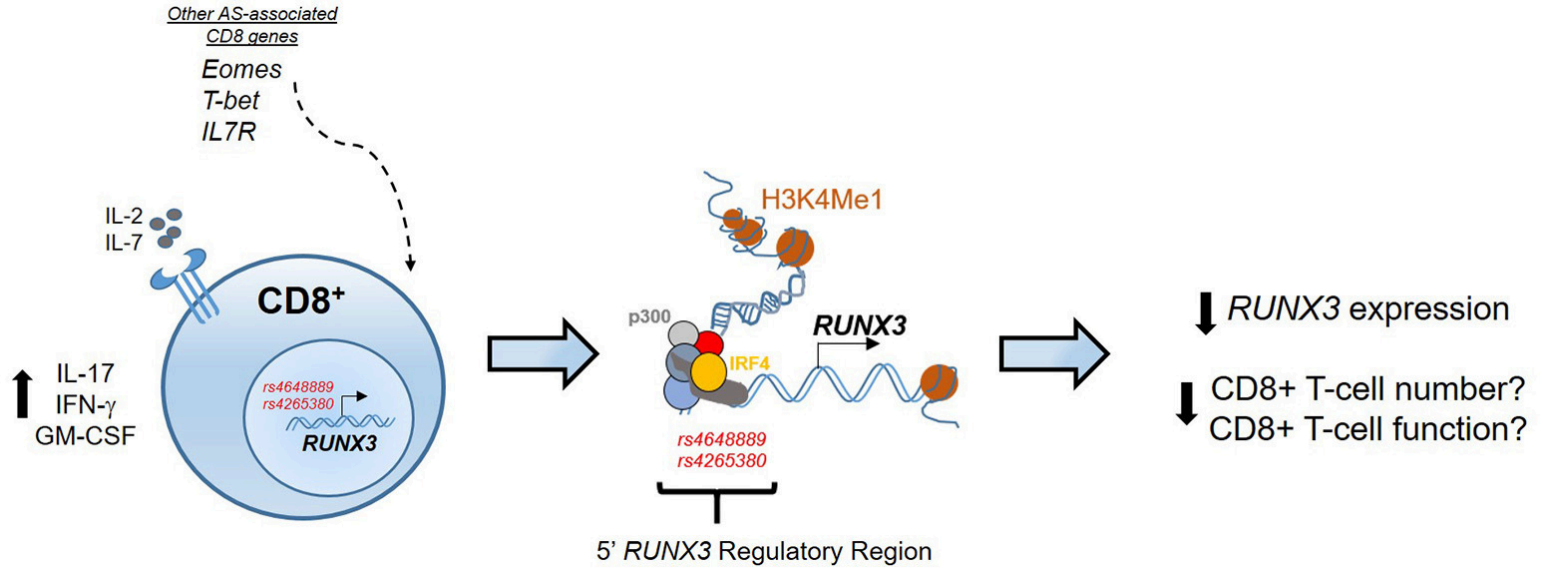
Epigenetic regulation at the RUNX3 AS-associated locus. In CD8+ T-cells the regulatory region upstream the promoter of RUNX3 is characterized by binding of several TFs, included p300 and IRF4, and enhancer histone marks (H3K4Me1). RUNX3 AS-risk allele has an epigenetic effect to reduce RUNX3 expression that might affect CD8+ T-cell numbers and function. The contribution of other genes associated with AS, like T-bet, Eomes and IL7R strengthen the involvement of CD8+ T-cells development pathway in AS pathogenesis.

Previously we have shown that an AS-associated SNP (*rs4648889*), located upstream of the *RUNX3* promoter affects RUNX3 gene expression in CD8+ T-cells through changes in transcription factor binding (58). These findings implicate CD8+ T-cells in the pathophysiology of AS, and raise the possibility that reduced CD8+ T-cell numbers and/or altered function may be contribute to its pathogenesis (Figure 1). Subsequently, we described another SNP (*rs4265380*) also upstream of *RUNX3* and only 500 bp from *rs4648889*, which has potential regulatory functions in monocytes rather than T-cells (59). The role of *RUNX3* in the myeloid compartment has not been extensively studied in immune biology (60, 61), but clearly could be very important in our understanding of the pathophysiology of AS. Similar genetic associations at the *RUNX3* locus have been also described in psoriatic arthritis, thereby revealing an unsurprising degree of genetic overlap between these two related forms of SpA (52).

Further evidence from Al-Mossawi and colleagues supports the plausible role of monocytes in AS pathogenesis. Their study showed that monocytes upregulate IL7R expression and soluble IL7R secretion after LPS treatment in a functional, genotype- and TNFα-dependant manner. These data draw attention to an unappreciated key myeloid role for AS risk variants at IL7R (62).

These findings suggest that the IL7R/RUNX3 axis might have a plausible role in monocyte biology and in the pathogenic process of AS, which up to now has been poorly investigated.

### TBX21

AS is associated with *rs11657479*, a SNP in the 3′ untranslated region (UTR) of *TBX21* (encoding the transcription factor T-bet) (10). Although the causal variant has not yet been defined since the locus has not been fine mapped. Expression of *TBX21* and T-bet is increased in patients with AS and homozygosity for the *rs11657479* risk allele increases T-bet expression. In the SKG mouse model of SpA, loss of T-bet expression protects against disease (63) suggesting that T-bet is an important regulator of immune responses in SpA. Genetic associations have also been reported at *TBX21* in AS and IBD (64), suggesting that T-bet may play an important role in these diseases, most likely involving control of mucosal barrier defenses.

T-bet was first described in CD4 T-cells as the key Th1 lineage-defining transcription factor controlling expression of IFN-γ and CXCR3 (65). It is now clear, however, that T-bet is widely expressed in immune cell subsets and controls functional differentiation of many cell types including CD8 T cells, NK cells, B cells.

Enhanced T-bet expression in AS is predominantly seen in CD8 T-cells and NK cells where it influences function of these cell types (63), both of which are strongly implicated in AS pathogenesis (66). The development and terminal maturation of immature NK cells, characterized by the expression of TNF-related apoptosis-inducing ligand (TRAIL) (67, 68), are dependent on T-bet (69). However, the importance of T-bet in defining NK cell biology may be tissue or context specific. For example in mice, liver resident NK cells (or ILC1 cells) depend on T-bet while conventional NK cells are strictly dependent on eomesodermin (67, 68).

T-bet regulation of CD8 T-cells responses is similarly context dependent. T-bet promotes differentiation of short-lived effector CD8 T-cells but does not influence long-lived memory CD8 T-cells (70). Importantly, T-bet cooperates with eomesodermin and other transcription factors to fine tune CD8 T-cell functions. For example, cooperative effects of T-bet and eomesodermin induce IFN-γ production by CD8 T cells (71). Indeed, in mice much is known about cooperation between T-bet and several transcription factors such as ZEB2 (72) and Blimp-1 (73). Yet our knowledge of cooperation between T-bet and other transcription factors in humans and in disease settings is limited. Given the genetic associations with *TBX21, EOMES*, and *RUNX3* in AS (10) further investigation of transcription factor biology in AS is warranted. More broadly, a better understanding of T-bet regulation of CD8 T cell responses in humans would be beneficial.

**Figure 2:**
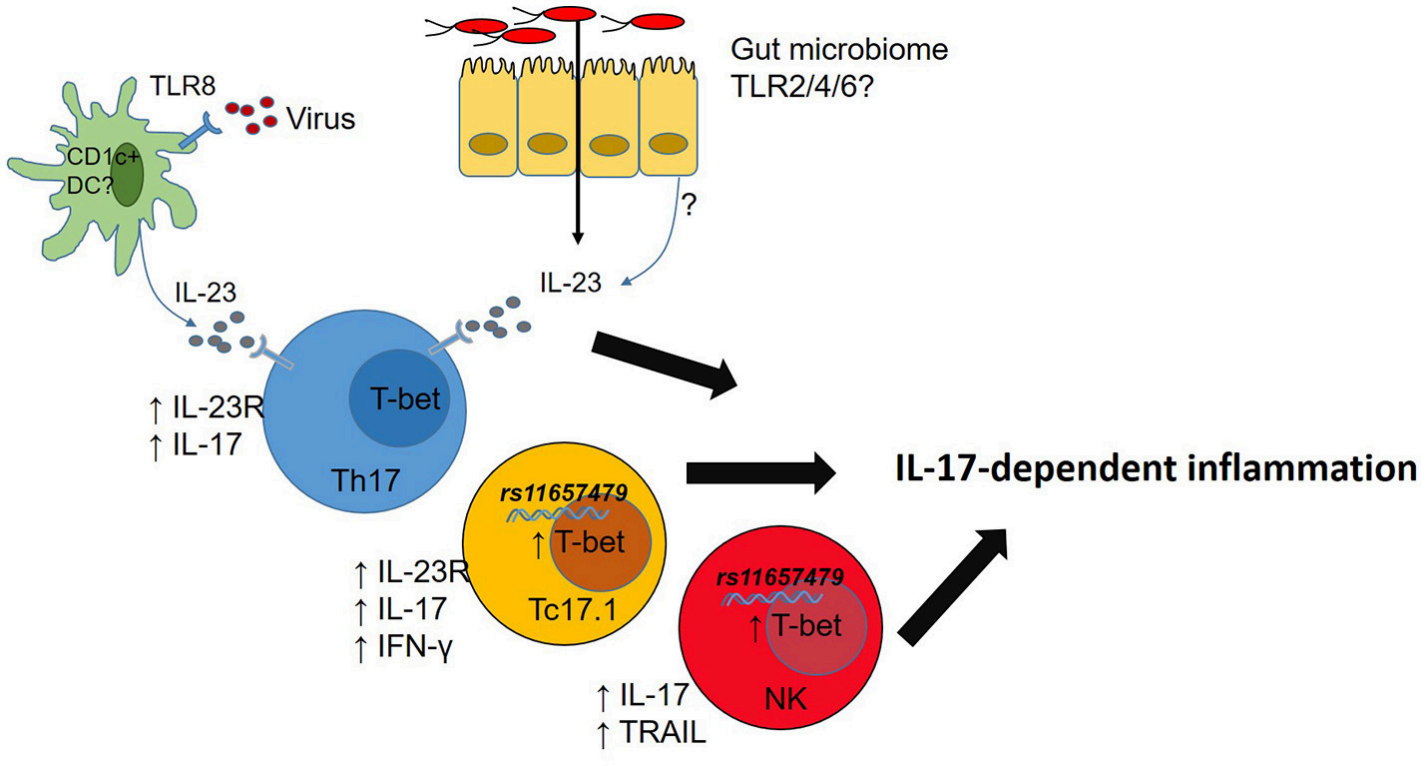
Microbial dysbiosis or viral infection drives IL-23 production. T-bet expression in T cells promotes IL-23R expression licensing IL-23-mediated inflammation. Carriage of rs11657479 enhances T-bet expression in CD8 T cells and NK cells and enhances pro-inflammatory functions in those cell types.

While studies in Tbx21^−/−^ mice have highlighted some important T-bet functions the regulation of immune cell responses by T-bet is nuanced. For example, graded expression of T-bet in CD4 T-cells responding to *Listeria monocytogenes* infections determines which cells produce IFN-γ (T-bet^hi^) and which ones do not mount a protective IFN- γ response (T-bet^lo^) (74). Of direct relevance to AS, IL-12 induces graded expression of T-bet in mouse CD8 T-cells which in turn controls functional potential of those CD8 T-cells (70) while T-bet expression CD4 Th17 cells stabilizes pathogenic Th17 cells (75). In mice at least, T-bet plays a role in controlling transcription of *Il23r* (76) and expression of *Cxcr3* in multiple immune cells (77), thereby shaping their tissue migratory potential. It is therefore clear that T-bet, alone and in concert with other transcription factors, fine tunes functions of multiple immune cell types but the relevance of this to chronic immune-mediated diseases in humans is yet to be investigated. Since T-bet also play important roles in gut barrier function (78, 79) dissecting the role T-bet plays in host-microbiome responses in chronic immune-mediated diseases is also likely to be of value to understanding disease- and tissue-specific immunopathogenesis processes (Figure 2).

## Coordinated Effects of AS-Associated Transcription Factors

There is still much to do to define all the functional consequences of AS-associated genetic variations in transcription factors like T-bet, eomesodermin and RUNX3. This is made more complicated by the fact that their effects are likely to be cell type specific.

It has been demonstrated that together with T-bet, RUNX3 regulates interferon-γ and interleukin 4 expression in T-helper Th1 cells. Moreover, in Th1 cells the Runx3 expression is induced in a T-bet-dependent manner (80). This fact implies a plausible synergy between these two factors and a plausible role in AS pathogenesis.

Although AS is highly heritable, the contribution of individual genes (or biological pathways) has been relatively poorly explored, with the notable exception of IL23-driven inflammation and a few other examples. The RUNX3/T-bet/eomesodermin axis could represent a key pathway for exploring the differentiation of many key immune cells relevant to AS (66).

The different examples we have here reported clearly illustrated how genetic associations can provide useful information about the finding and the development of potential drug targets.

Of note, the interplay between chromatin state, TF occupancy and tissue-specific gene expression is critical (81), and an integrative approach is needed in order to increase the power of the analysis and fully understand the AS-associated regulatory mechanism, of which the RUNX3/T-bet axis is not exempt.

## Next Steps in RUNX3 and T-Bet Biology in AS

Current data support the importance of RUNX3+ CD8 T-cells, T-bet+ CD8 T-cells, and T-bet+ NK cells in the pathogenesis of AS. These data in turn validate the need to study these immune cell populations in further detail in AS cases. The functional properties of RUNX3+ CD8 T-cells and T-bet+ CD8 T-cells and NK cells are still largely unresolved. The effects of manipulating these cell populations in mice and humans requires further investigation. A detailed functional genomic approach, such as cell-type specific ChIP-Seq and RNA-seq, will help to identify the downstream targets of RUNX3 and T-bet in these cells. Only through these more comprehensive studies will we be able to define useful therapeutic targets more accurately.

## Conclusions and Perspectives

GWAS have the potential to provide key insights into the pathogenesis of complex polygenic disorders even where the strength of individual associations seem weak. Even modest genetic associations may reveal therapeutic targets with profound biological effects. Thus, the relatively modest but robust association of the R381N SNP in the cytoplasmic tail of IL-23R, which has functional consequences, clearly implicates IL-23 pathways in AS; targeting the main effector cytokine IL-17A in this pathway with the therapeutic antibody secukinumab has proved highly effective (35).

Nevertheless, considerable work will be required to finely dissect the biological mechanisms at play to elucidate why, for example, anti-IL-23 antibodies work in IBD and psoriasis but not in AS (38–40). It will be also vital to see the results of the ongoing trials targeting GPR65, a G-protein coupled receptor overexpressed in AS patients, with an antibody against GM-CSF: a positive outcome will open new ways for future investigations.

In general, such coding polymorphisms are unusual in the context of polygenic diseases and more subtle genetic influences, arising from polymorphisms in regulatory elements influencing gene expression, are more likely to be implicated in complex diseases like AS (82). For example, additive influences have been described between the various genes in the IL-23 signaling pathway on the effector functions of Th1 and Th17 cells in patients with SpA (83), but the full range of factors involved in the regulation of Th17 cells is only just beginning to be appreciated (84). This is the goal also for the RUNX3/T-bet/eomesodermin axis. Targeting these transcription factors, such as RUNX3, directly will not only be technically challenging but may prove harmful due to their broad effects on cell biology and human immunity. However, determining the interplay between them and defining their targets, both individual and collective, in the context of AS will advance our understanding of the pathogenesis of AS. Ultimately this may identify novel cell-specific therapeutic targets with acceptable risk benefit profiles.

## Author Contributions

MV, AR, PW, and TK conceived the manuscript. MV, PW, and TK drafted the manuscript and all the authors revised the final version prior submission.

### Conflict of Interest Statement

The authors declare that the research was conducted in the absence of any commercial or financial relationships that could be construed as a potential conflict of interest.
